# Parental reflexivity and latent psychological violence among urban parents in Kazakhstan and Kyrgyzstan: an exploratory study

**DOI:** 10.3389/fpsyg.2026.1935605

**Published:** 2026-09-30

**Authors:** Maigul Bagibayeva, Mariyam Bapayeva, Farida Kuantkanova, Marhabbat Nurov

**Affiliations:** 1 Department of Psychology, Kazakh National Women’s Teacher Training University, Almaty, Kazakhstan; 2 Higher School of International Relations and Diplomacy, Turan University, Almaty, Kazakhstan

**Keywords:** Central Asia, family-of-origin violence, fatherhood, father–mother comparisons, Kazakhstan and Kyrgyzstan, latent psychological violence, parental reflexivity, uyat

## Abstract

**Introduction:**

Parental reflective functioning (PRF) has been associated with less harmful parenting behavior and a lower likelihood of intergenerational continuity of violence. However, little is known about whether the association between parental reflexivity and psychologically harmful parenting differs between fathers and mothers in Central Asia. This study examined parental reflexivity, measured using A. V. Karpov’s Reflexivity Scale as an operational proxy for PRF, and its association with latent psychological violence among urban parents in Kazakhstan and Kyrgyzstan.

**Methods:**

A cross-sectional multi-city study was conducted in Almaty, Shymkent, Turkestan, and Bishkek. The analytical sample (*N* = 400), drawn from a larger dataset (*n* = 500), included 158 fathers and 242 mothers. Measures included reflexivity, aggression, empathy, latent psychological violence (ILV), uyat intensity, and family-of-origin violence. Analyses comprised parent-group-specific regressions, bootstrap coefficient comparisons, hierarchical moderation models with a PRF × parent-group interaction, and robustness analyses using recruitment-site fixed effects, robust standard errors, the full sample, parent-group weighting, and additional family-context covariates.

**Results:**

Reflexivity was strongly inversely associated with latent psychological violence in both parent groups. The PRF–ILV slope was descriptively steeper among fathers than mothers (*b* = −0.900, *R*^2^ = 0.485 vs. *b* = −0.779, *R*^2^ = 0.417), but the difference was not statistically significant (*z* = −1.27, *p* = 0.204; 95% bootstrap CI [−0.320, 0.067]). The PRF × parent-group interaction was also nonsignificant (Δ*R*^2^ = 0.002, *p* = 0.201). Among highly reflective parents with family-of-origin violence, none of five fathers met the exploratory high-ILV threshold (ILV ≥ 70), compared with 1 of 7 mothers; this difference was nonsignificant (*p* = 1.000). Family-of-origin violence was associated with lower reflexivity among both fathers (*d* = 0.64) and mothers (*d* = 0.74).

**Discussion:**

Parental reflexivity showed a robust inverse association with latent psychological violence, but formal analyses provided no evidence that this association differed between fathers and mothers. The descriptively steeper father association should therefore be regarded as hypothesis-generating rather than as evidence of a father-specific effect. These findings support further longitudinal and intervention research in urban Central Asian populations.

## 1 Introduction

The problem of intergenerational continuity of violence remains an important issue in clinical and family psychology (Cicchetti and Toth, 2005; Fonagy and Target, 1997). Parental reflective functioning (PRF)—the capacity to understand one’s own and one’s child’s behavior in terms of underlying mental states, needs, and intentions—has been conceptualized as one mechanism linking parents’ internal representations to parenting behavior and parent–child relationships (Fonagy et al., 2002; Slade, 2005). Empirical research on parental reflective functioning has historically concentrated primarily on mothers, whereas father-specific evidence remains comparatively limited (Stover and Kiselica, 2014; Buttitta et al., 2019).

The Central Asian setting is analytically important because family roles are shaped by a combination of post-Soviet institutional legacies and persistent gendered expectations. Evidence from Kazakhstan documents a continuing imbalance in the division of housework and childcare despite high levels of women’s participation in education and employment (Kan, 2023), while comparative work on Kyrgyzstan describes changes in parental roles against a background of enduring traditional family structures (Wejnert and Djumabaeva, 2005). Recent research in Kazakhstan has also shown that parental attitudes and family dynamics vary across sociodemographic contexts, including region, marital status, and family structure (Bapayeva et al., 2026). Against this heterogeneous background, whether parental reflexivity is differently associated with latent psychological violence among fathers and mothers remains insufficiently examined.

The present study aims to address this gap. We pose three interrelated research questions: (1) whether parental reflexivity is associated with latent psychological violence among fathers and mothers and whether the magnitude of this association differs statistically between the two parent groups; (2) which psychological, cultural, and sociodemographic characteristics are associated with parental reflexivity, with particular exploratory attention to fathers; and (3) how high levels of latent psychological violence are distributed descriptively across levels of reflexivity among parents reporting a history of violence in the family of origin. The study is explicitly exploratory and does not assume *a priori* that parent group moderates the association between parental reflexivity and latent psychological violence.

## 2 Theoretical foundations and literature review

### 2.1 Reflective functioning: the construct and its operationalization

The construct of parental reflective functioning was systematically operationalized by Fonagy and Target (1997) and Fonagy et al. (2002) as the capacity to perceive one’s own behavior and the behavior of the child in terms of mental states—desires, beliefs, feelings, and intentions. Rooted in Bowlby’s theory of internal working models (Bowlby, 1969) and further developed within the mentalization-based approach (Slade, 2005; Bateman and Fonagy, 2004), this construct has been linked to the quality of parent–child interaction. A systematic review found that parental reflective functioning is associated with observed parenting behavior, particularly sensitivity, although the strength and consistency of associations vary across measures and samples (Stuhrmann et al., 2022). Father-focused research likewise suggests that paternal reflective functioning may be associated with emotionally supportive parenting, without establishing a universally stronger effect among fathers (Buttitta et al., 2019).

Within the Russian psychological tradition, the closest operational analog is reflexivity as conceptualized by Karpov (2003)—an integrative metacognitive capacity involving reflection on current activity, past experience, and prospective action. Although conceptually related to reflective functioning at the level of metacognitive self-monitoring and perspective taking, Karpov’s construct is broader and should not be treated as equivalent to child-specific parental reflective functioning. In the present parenting context, it is used to index a general reflexive capacity that may be relevant to how parents monitor and interpret their own responses.

This terminological clarification is essential for the correct interpretation of the findings. A. V. Karpov’s Reflexivity Scale measures general metacognitive reflexivity and is not a specialized instrument for assessing parental reflective functioning, such as the PRFQ (Luyten et al., 2017). Therefore, throughout all sections of the present article, the abbreviation PRF is used as a conventional designation for parental reflexivity, which serves as an operational proxy indicator of reflective functioning, while substantive conclusions are formulated with reference to reflexivity as a broader construct. This limitation of operationalization is taken into account in the interpretation of the results and is discussed in Section 6.

### 2.2 Gender, socialization, and reflective competence

Theoretical models of emotion socialization propose that emotion-related socialization can vary by gender, while also emphasizing substantial situational, individual, and cultural variation and cautioning against exaggerated gender stereotypes (Brody, 1997; Eisenberg et al., 1998). Traditional masculinity norms may, in some contexts, discourage emotional expression and professional help-seeking (Levant, 1992; Addis and Mahalik, 2003). These propositions provide a theoretical rationale for examining father–mother patterns, but they do not imply that such differences are universal or present in the current sample.

In the Central Asian context, a relevant cultural concept is uyat, understood here as a culturally codified form of social shame. Scholarship on Central Asia describes uyat as a mechanism involved in the regulation of social interactions and gendered norms (Thibault and Caron, 2022). Recent qualitative research in Kazakhstan has also documented the role of uyat in survivors’ responses to intimate partner violence and in interactions with professionals (Akhmetova et al., 2025). These culturally situated processes may intersect with broader masculinity-related barriers to help-seeking (Addis and Mahalik, 2003), but the present study does not directly measure help-seeking behavior.

### 2.3 The post-Soviet context: masculinity, fatherhood, and psychological culture

Within post-Soviet Central Asia, parenting roles should not be treated as uniform. Evidence indicates that traditional divisions of household and childcare responsibilities remain salient while also varying across families and social contexts (Kan, 2023; Wejnert and Djumabaeva, 2005). These contextual patterns motivate a father-inclusive analysis but do not, by themselves, establish father-specific psychological mechanisms.

The centrality of the family as an institution in the formation of Kazakhstani identity has been noted in prior scholarship (Seitakhmetova and Zhandossova, 2019). Religious revival has also reinforced selected traditional patterns in family and marital relations in Kazakhstan, with Islamic norms increasingly informing expectations around marriage, family authority, and gender roles (Zhandossova et al., 2023). Normative regulation extends beyond the domestic sphere into public expressions of identity. Research on Kazakh women’s digital religious practices shows that visual self-presentation on Instagram emerges through an interplay of Islamic norms, traditional cultural codes, and public visibility (Toktarbekova et al., 2025), while comparative analysis of state counter-extremism frameworks illustrates how religious expression, belonging, and social legitimacy are also institutionally negotiated (Seitakhmetova and Zhandossova, 2026). Taken together, these studies point to a layered normative environment spanning family, religion, and public identity. In the present study, this literature is used as contextual background for interpreting parental reflexivity, particularly in Kazakhstan, rather than as evidence of a father-specific psychological mechanism or as a basis for generalizing Kazakhstan-specific processes to Kyrgyzstan.

Research on reflective functioning has historically focused primarily on mothers, while father-specific studies remain comparatively limited (Stover and Kiselica, 2014). Existing father-focused work indicates that paternal reflective functioning can be associated with emotionally supportive and autonomy-supportive parenting behaviors, although findings remain limited and context dependent (Buttitta et al., 2019). The present study therefore treats fathers as an analytically important but not presumed-different parent group, particularly in a setting where parental attitudes vary across sociodemographic and family conditions (Bapayeva et al., 2026).

### 2.4 Research hypotheses

*H1*: The regression coefficient for the association PRF → ILV is expected to be descriptively more pronounced in the father subsample than in the mother subsample, with a comparable proportion of explained variance. Formal testing of coefficient equality and of the PRF × parent-group interaction is conducted in an exploratory mode, without claiming established moderation.

*H2*: A history of violence in the family of origin (FOV) is expected to be associated with lower parental reflexivity among both fathers and mothers. The question of whether the magnitude of this association differs between the two parent groups is treated as an open exploratory issue.

*H3*: Among parents with a history of violence in the family of origin (FOV), the frequency of scores meeting the operational high-ILV threshold (ILV ≥ 70) is expected to be descriptively lower in the highly reflective father subgroup (PRF > 70) than in the corresponding mother subgroup. Because the ILV threshold is used for exploratory categorization and the relevant cells are very small, this expectation is treated as a preliminary descriptive observation rather than evidence of a clinical cutoff or intergenerational transmission effect.

*H4*: The intensity of uyat is expected to show a stronger negative association with parental reflexivity among fathers than among mothers. This parent-group difference is tested exploratorily rather than treated as an established gender-specific mechanism.

All four hypotheses are formulated within the logic of an exploratory study: their purpose is to structure the description of empirical regularities rather than to provide a basis for confirmatory conclusions regarding parent-group moderation of the association between parental reflexivity and latent psychological violence.

## 3 Materials and methods

### 3.1 Sample and procedure

The original dataset was collected as part of a cross-sectional, urban multi-city study (*n* = 500 parents; Almaty, Shymkent, and Turkestan, Kazakhstan; Bishkek, Kyrgyzstan; September 2024–February 2025). All four recruitment sites were urban administrative centers. From this dataset, an analytical subsample of *N* = 400 was formed through stratified selection: it retained all 158 fathers and included 242 mothers selected using a pseudo-random algorithm with a fixed seed (seed = 2024), ensuring reproducibility. Stratification by parent group, with guaranteed inclusion of the entire father subsample, was chosen to maximize statistical power for father–mother comparisons while reducing the imbalance in group sizes.

The decision to form an analytical subsample of *N* = 400 was driven by the need to improve the balance between the father and mother groups while fully retaining the father cohort. Because excluding a portion of the mother observations could affect the estimates, all key models were re-estimated in the full sample of *n* = 500—without excluding any observations and also with post-stratification weighting by parent group—as part of the sensitivity analysis (section 4.8).

The inclusion criteria were biological parenthood of at least one child aged 3–16 years, co-residence with the child at the time of the study, and written voluntary informed consent to participate. The study protocol was approved by the Research Ethics Committee of the Kazakh National Women’s Teacher Training University (Almaty, Kazakhstan; Protocol No. IRB-2024/09-KZ); all participants provided written voluntary informed consent. The sociodemographic composition of the analytical subsample is presented in Table 1. In total, 297 participants were recruited at the three Kazakhstan sites and 103 in Bishkek, Kyrgyzstan. The mean age was 35.8 years (SD = 6.8) among fathers and 35.1 years (SD = 6.7) among mothers; 55.7% of fathers and 49.6% of mothers had completed higher education; and 60.1% of fathers and 59.9% of mothers were in a registered marriage. No statistically significant father–mother differences were detected in the recorded sociodemographic characteristics (all *p* ≥ 0.264).

**Table 1:** Socio-demographic characteristics of the analytical subsample by parent group (*N* = 400).

Characteristic	Fathers (*n* = 158)	Mothers (*n* = 242)	Total (*N* = 400)	Test	*p*
Age, years: M (SD)	35.8 (6.8)	35.1 (6.7)	35.4 (6.7)	*t* = 1.12	0.264
Age, years: median (range)	36 (21–54)	35 (21–54)	35 (21–54)		
Education, *n* (%)				χ^2^(3) = 3.31	0.346
General secondary	9 (5.7)	19 (7.9)	28 (7.0)		
Specialized secondary	33 (20.9)	45 (18.6)	78 (19.5)		
Incomplete higher	28 (17.7)	58 (24.0)	86 (21.5)		
Higher	88 (55.7)	120 (49.6)	208 (52.0)		
Marital status, *n* (%)				χ^2^(4) = 2.33	0.675
Married (registered)	95 (60.1)	145 (59.9)	240 (60.0)		
In partnership	27 (17.1)	31 (12.8)	58 (14.5)		
Divorced	22 (13.9)	43 (17.8)	65 (16.3)		
Widowed	5 (3.2)	10 (4.1)	15 (3.8)		
Single	9 (5.7)	13 (5.4)	22 (5.5)		
Recruitment site, *n* (%)				χ^2^(3) = 0.84	0.840
Almaty, Kazakhstan	60 (38.0)	85 (35.1)	145 (36.3)		
Shymkent, Kazakhstan	35 (22.2)	63 (26.0)	98 (24.5)		
Turkestan, Kazakhstan	22 (13.9)	32 (13.2)	54 (13.5)		
Bishkek, Kyrgyzstan	41 (25.9)	62 (25.6)	103 (25.8)		
Number of children					
M (SD)	2.07 (0.88)	2.04 (0.87)	2.05 (0.87)	*t* = 0.32	0.752
One child, *n* (%)	43 (27.2)	68 (28.1)	111 (27.8)		
Two children, *n* (%)	73 (46.2)	115 (47.5)	188 (47.0)		
Three children, *n* (%)	30 (19.0)	40 (16.5)	70 (17.5)		
Four children, *n* (%)	12 (7.6)	19 (7.9)	31 (7.8)		
Self-rated family income, *n* (%)				χ^2^(2) = 0.92	0.631
Below average	36 (22.8)	65 (26.9)	101 (25.3)		
Average	85 (53.8)	126 (52.1)	211 (52.8)		
Above average	37 (23.4)	51 (21.1)	88 (22.0)		

Percentages are column percentages within each parent group. Continuous variables were compared using independent-samples *t*-tests, and categorical variables were compared using χ^2^ tests. In regression models, education was entered as an ordinal variable (1 = general secondary, 2 = specialized secondary, 3 = incomplete higher, 4 = higher education). Family income was assessed on a three-category self-rating scale; monetary income was not collected. Child age and sex were not available as variables in the analytical dataset.

The analytical comparisons are between participants recorded in the dataset as fathers and mothers. The manuscript therefore refers to these two parent groups and does not generalize the findings to gender identities that were not represented as separate analytic categories.

All measures were administered in writing as a single questionnaire booklet. At the three Kazakhstan sites (Almaty, Shymkent, and Turkestan), participants could complete either the Russian-language or the Kazakh-language version of the battery; in Bishkek, the Russian-language version was used. A Kyrgyz-language version was not prepared. The Karpov Reflexivity Scale and the Yusupov Empathy Diagnostic Method were administered in their original Russian versions, and the Buss–Perry Aggression Questionnaire was administered in its published Russian-language adaptation. The author-developed ILV and UIS were drafted in Russian. Kazakh versions were prepared by bilingual members of the research team and independently back-translated into Russian by a bilingual psychologist; the source and back-translated versions were compared for conceptual equivalence, and wording was refined before pilot testing. Because the language version selected by each participant was not stored as a variable in the analytical dataset, measurement invariance across language versions could not be tested; this limitation is discussed in section 6.

Where questionnaire items referred to a child, the wording used “my child.” The instructions did not specify a single target child. Parents with one eligible child answered with that child in mind, whereas parents with more than one child aged 3–16 years were instructed to describe their usual behavior toward their children without selecting one child. Accordingly, the ILV total score reflects typical parenting behavior within the family rather than behavior toward one identified child; this design feature is treated as a limitation. The questionnaire recorded the number of children and, in free text, the ages of co-resident children, but the analytical dataset did not contain child-level age or sex variables. These characteristics therefore could not be entered as covariates (section 3.3).

### 3.2 Measurement instruments

Parental reflexivity, used as an operational proxy for parental reflective functioning (PRF), was assessed with an adapted 27-item Karpov Reflexivity Scale (Karpov, 2003), with scores ranging from 27 to 135; α = 0.89. The Inventory of Latent Violence (ILV) is an author-developed 54-item instrument covering six domains of covert psychological violence: guilt manipulation, emotional rejection, public comparison and shaming, threat of withdrawal of love, gaslighting, and discipline through shame; α = 0.81. The Yusupov Empathy Diagnostic Method (Yusupov, 1995) comprised 36 items; α = 0.84. The Buss–Perry Aggression Questionnaire (Buss and Perry, 1992) was used in its Russian-language adaptation and comprised 24 items; α = 0.81. The Uyat Intensity Scale (UIS) is an author-developed 12-item instrument; α = 0.78. Experience of violence in the family of origin (FOV) was recorded using a binary self-report item supplemented by four severity-specifying subitems.

Each ILV item describes a specific parental behavior (for example, “I remind my child how much I do for him when he disappoints me”; “I criticize my child in front of others”; “I say, ‘What will people think if they find out?’”). Responses were recorded on a three-point frequency scale: 1 = never, 2 = rarely, and 3 = sometimes/often. The six domains contain nine items each: items 1–9, guilt manipulation; 10–18, emotional rejection and silent treatment; 19–27, public comparison and shaming; 28–36, threats of withdrawal of love; 37–45, gaslighting; and 46–54, discipline through shame. All items are scored in the same direction; no reverse-scored items are used. The total score is the unweighted sum of all 54 responses and can theoretically range from 54 to 162. Each domain score is the sum of its nine items and ranges from 9 to 27. Questionnaires were checked for completeness at collection, and the analytical dataset contained no missing ILV item responses; therefore, no imputation or prorating rule was required.

The ILV ≥ 70 threshold was not derived from an external clinical standard; no clinical cutoff has been established for this author-developed instrument. The research team specified 70 as an operational threshold before conducting the subgroup comparisons. A score of 70 is 16 points above the scale minimum and corresponds to a mean item response of approximately 1.30; for illustration, it could result from approximately 16 of the 54 behaviors being reported at least “rarely” if the remaining behaviors were reported as “never.” In the analytical subsample, the threshold lies at approximately the 62nd percentile of the ILV distribution, with 38.5% of participants scoring 70 or higher. It is therefore treated strictly as an exploratory high-score threshold rather than a diagnostic criterion. Sensitivity checks using alternative thresholds are reported in section 4.6.

For the threshold-based exploratory analyses reported below, ILV ≥ 70 is treated strictly as an operational high-score threshold. It is not presented as a clinically validated diagnostic cutoff. Because FOV history and current ILV were assessed within a cross-sectional self-report design, the co-occurrence of FOV = yes and ILV ≥ 70 is described as a high-ILV frequency rather than as a direct measure of intergenerational transmission.

#### 3.2.1 Psychometric validation of the authors’ instruments (ILV and UIS)

Because the Inventory of Latent Violence (ILV) served as the dependent variable, and the Uyat Intensity Scale (UIS) as a key cultural mechanism, the authors’ instruments underwent extended psychometric validation. The full sample (*n* = 500) was randomly divided into two equal halves (250 observations each; seed = 2024). In the first half, exploratory factor analysis was conducted using principal axis factoring with promax rotation. The Kaiser–Meyer–Olkin measure of sampling adequacy was 0.91, and Bartlett’s test of sphericity was significant at *p* < 0.001. A six-factor solution, substantively corresponding to the six *a priori* domains of the ILV—guilt manipulation, emotional rejection, public comparison and shaming, threat of love withdrawal, gaslighting, and discipline through shame—explained 58.3% of the total variance, with item factor loadings ranging from 0.42 to 0.81 and no pronounced cross-loadings (> 0.30).

In the second half of the sample, confirmatory factor analysis using MLR estimation confirmed the six-factor structure: χ^2^/df = 1.86; CFI = 0.941; TLI = 0.936; RMSEA = 0.044 (90% CI [0.040; 0.048]); SRMR = 0.051—values consistent with commonly accepted criteria for acceptable model fit (Hu and Bentler, 1999). The reliability of the ILV subscales ranged from α = 0.74 to 0.86 (McDonald’s ω = 0.76–0.88), while the full scale showed α = 0.81. The unidimensional UIS model also demonstrated an acceptable fit to the data: CFI = 0.958; RMSEA = 0.052; SRMR = 0.041; α = 0.78. Test–retest reliability, assessed in a subsample of 62 participants over a three-week interval, was *r* = 0.84 for the ILV and *r* = 0.79 for the UIS.

Convergent validity was supported by the theoretically expected configuration of associations: the total ILV score correlated positively with the aggression index (*r* = 0.585; *p* < 0.001) and negatively with empathy (*r* = −0.553; *p* < 0.001) and reflexivity (*r* = −0.661; *p* < 0.001). The UIS score was positively associated with the ILV (*r* = 0.443; *p* < 0.001) and negatively associated with reflexivity (*r* = −0.476; *p* < 0.001). A comparable correlational structure was reproduced separately in the father subsample (ILV–AI *r* = 0.594; ILV–EMP *r* = −0.559) and the mother subsample (*r* = 0.577 and *r* = −0.548, respectively), indicating the stability of the instruments’ nomological network.

#### 3.2.2 Measurement invariance across father and mother groups

The validity of comparisons between fathers and mothers was examined using multigroup confirmatory factor analysis (Cheung and Rensvold, 2002; Putnick and Bornstein, 2016). For the ILV, the configural model showed acceptable fit (CFI = 0.936; RMSEA = 0.046). Constraining factor loadings to equality produced only small changes in fit (metric invariance: ΔCFI = −0.004; ΔRMSEA = +0.001), supporting comparison of structural associations such as regression coefficients across father and mother groups. Full scalar invariance was not obtained for the ILV. Partial scalar invariance was achieved after freeing the intercepts of two items from the “public comparison and shaming” domain (ΔCFI = −0.006): item 22 (“I tell my relatives or neighbors about my child’s misdeeds”) and item 27 (“I openly state that another child in the family is better”). Within this domain, these two items showed the largest father–mother mean differences in opposite directions (fathers lower on item 22 and higher on item 27), a pattern consistent with localized intercept noninvariance. Accordingly, ILV mean comparisons should be interpreted more cautiously than comparisons of associations because not all item intercepts were invariant. For the UIS, full scalar invariance was established (ΔCFI = −0.003), supporting both structural and mean-level comparisons across the two parent groups. The ΔCFI ≤ 0.01 criterion was used as the primary decision rule (Cheung and Rensvold, 2002).

The fit indices shown in Table 2 are those preserved in the archived multigroup analysis output. The complete sequence of χ^2^ values and degrees of freedom for each nested step, together with TLI and SRMR, was not retained in a form that could be reproduced reliably and therefore was not reconstructed. Invariance decisions were based on ΔCFI < 0.01 (Cheung and Rensvold, 2002), supported by ΔRMSEA < 0.015 (Chen, 2007); the reported changes were within these limits at each available step.

**Table 2:** Summary of measurement-invariance results reported in the current analysis output.

Instrument	Model	Constraint	Reported fit/change	Interpretation
ILV	Configural	Same factor structure; parameters freely estimated	CFI = 0.936; RMSEA = 0.046	Common factor structure supported
ILV	Metric	Factor loadings constrained equal	ΔCFI = −0.004; ΔRMSEA = +0.001	Structural associations may be compared
ILV	Partial scalar	Loadings equal; intercepts equal except items 22 and 27 (“public comparison and shaming”)	ΔCFI = −0.006	Mean comparisons require caution; two intercepts noninvariant
UIS	Full scalar	Loadings and intercepts constrained equal	ΔCFI = −0.003	Structural and mean-level comparisons supported

ΔCFI and ΔRMSEA values are reported relative to the preceding, less constrained model. Only fit/change statistics preserved in the archived analysis output are shown; χ^2^, df, TLI, and SRMR for the nested models were not available and were not reconstructed. Items 22 and 27 are the two ILV items whose intercepts were freely estimated across parent groups in the partial scalar model.

### 3.3 Analytic strategy

Regression, bootstrap, and sensitivity analyses were conducted in Python 3.11 using the NumPy, SciPy, and StatsModels libraries. Descriptive comparisons were accompanied by Cohen’s d. Separate simple regressions of ILV on parental reflexivity were estimated for fathers and mothers, and the difference between the two slope coefficients was tested using the Paternoster–Edes procedure (Paternoster et al., 1998). A pooled hierarchical model was subsequently used to test the PRF × parent-group interaction. Age and education were included as a deliberately parsimonious set of basic parent-level demographic covariates in the primary analytical model; this adjustment should not be interpreted as sufficient control for all potential confounding. PRF was mean-centered before construction of the interaction term. The parent-group indicator was coded father = 1 and mother = 0. In the fixed-effects specification, Almaty was the reference recruitment site.

Model 1 (father- and mother-specific regression): ILVᵢ = β₀g + β₁g(PRFᵢ) + εᵢ, g ∈ {father, mother}.

Model 2 (pooled interaction model): ILVᵢ = β₀ + β₁(PRFᶜᵢ) + β₂(ParentGroupᵢ) + β₃ (PRFᶜᵢ × ParentGroupᵢ) + εᵢ.

Model 3 (extended specification): ILVᵢ = β₀ + β₁(PRFᶜᵢ) + β₂(ParentGroupᵢ) + β₃(UISᵢ) + β₄(FOVᵢ) + β₅(Empathyᵢ) + β₆(Ageᵢ) + β₇(Educationᵢ) + β₈ (PRFᶜᵢ × ParentGroupᵢ) + Σⱼδⱼ(Siteⱼᵢ) + εᵢ.

Here, ILV is the dependent variable; PRFᶜ denotes centered parental reflexivity; ParentGroup is coded father = 1 and mother = 0; FOV is the binary family-of-origin violence indicator; and Site represents recruitment-site fixed effects, with Almaty as the reference. The interaction coefficient β₈ tests whether the PRF–ILV slope differs between fathers and mothers.

The source dataset also contained marital status (married, in partnership, divorced, widowed, or single), self-rated family income (below average, average, or above average), number of children, age, education, and recruitment site. Child age and sex were not available as variables in the analytical dataset (section 3.1). To examine whether the parsimonious covariate set in Model 3 left relevant family-context differences insufficiently controlled, an additional sensitivity specification (Model 4) added registered marital status (registered marriage = 1; all other statuses = 0), family income (two dummy variables with “average” as the reference category), and number of children to Model 3 while retaining the PRF × parent-group interaction and recruitment-site fixed effects. A separate specification coded all five marital-status categories individually. Results are reported in section 4.8 (Table 3).

**Table 3:** Sensitivity model with additional family-context covariates (Model 4) predicting ILV (*N* = 400).

Predictor	*b*	SE	*t*	*p*	95% CI for b
PRF (centered)	−0.53	0.07	−7.09	<0.001	[−0.67; −0.38]
Parent group (father = 1)	−2.84	1.61	−1.76	0.078	[−6.00; 0.32]
PRF × Parent group	−0.15	0.09	−1.57	0.117	[−0.33; 0.04]
Uyat Intensity (UIS)	0.31	0.10	3.22	0.001	[0.12; 0.50]
FOV (0/1)	5.01	1.69	2.97	0.003	[1.69; 8.33]
Empathy (EMP)	−0.16	0.05	−2.94	0.003	[−0.27; −0.05]
Age	0.12	0.12	0.99	0.321	[−0.11; 0.35]
Education (1–4)	−0.23	0.80	−0.29	0.769	[−1.80; 1.33]
Married (registered marriage = 1)	0.94	1.60	0.59	0.557	[−2.20; 4.08]
Income: above average (ref. average)	2.87	1.99	1.44	0.152	[−1.06; 6.79]
Income: below average (ref. average)	−0.71	1.89	−0.38	0.706	[−4.42; 3.00]
Number of children	−1.71	0.90	−1.90	0.059	[−3.48; 0.06]
Recruitment site: Bishkek (ref. Almaty)	0.90	2.02	0.45	0.656	[−3.07; 4.86]
Recruitment site: Shymkent	1.68	2.03	0.83	0.407	[−2.30; 5.66]
Recruitment site: Turkestan	2.20	2.48	0.89	0.376	[−2.68; 7.07]

*R*^2^ = 0.503; adjusted *R*^2^ = 0.483; *F*(15, 384) = 25.9, *p* < 0.001. Change relative to the recruitment-site fixed-effects specification in Table 6, Step 4: Δ*R*^2^ = 0.009; *F*(4, 384) = 1.66, *p* = 0.159. PRF was mean-centered before construction of the interaction term. Ordinary least squares estimates are shown; HC3 robust standard errors yielded p = 0.138 for the interaction and did not alter the overall significance pattern.

To increase the robustness of the estimates, the analytical strategy was supplemented with the following procedures. First, the statistical significance of differences in regression coefficients between the father and mother subsamples was assessed not only using the slope-difference test (Paternoster et al., 1998), but also by stratified bootstrapping (5,000 replications, percentile-based 95% confidence intervals; seed = 2024) (Efron and Tibshirani, 1993). Second, recruitment-site fixed effects were added, with Almaty as the reference category, and all models were replicated using HC3 robust standard errors. These fixed effects adjust for observed differences among the four recruitment sites; however, they do not identify a separate country effect because Kyrgyzstan is represented only by Bishkek, whereas Kazakhstan is represented by three cities. Third, 95% Wilson confidence intervals were calculated for proportions in small cells of the factorial matrix. Fourth, the key models were re-estimated in the full sample of *n* = 500, both without adjustment and using weighted least squares with post-stratification weights by parent group. Finally, given the exploratory nature of the study, the results are interpreted cautiously; no correction for multiple comparisons was applied, which limits the strength of confirmatory inference.

## 4 Results

### 4.1 Descriptive characteristics of the study variables

Table 1 summarizes the sociodemographic composition of the two parent groups. No statistically significant father–mother differences were detected in age, education, marital status, recruitment site, number of children, or self-rated family income (all *p* ≥ 0.264). These findings do not establish equivalence between the groups, but they indicate no marked imbalance in the recorded sociodemographic characteristics. Table 4 presents descriptive statistics for the principal psychological study variables by parent group. None of the pairwise comparisons reached the conventional threshold of statistical significance, and the observed effect sizes were small (|*d*| < 0.17 for all continuous variables). Thus, no statistically detectable father–mother differences were observed in mean reflexivity, aggression, uyat intensity, or FOV frequency in the present sample. These nonsignificant findings should not be interpreted as evidence of equivalence.

**Table 4:** Descriptive statistics for the key study variables by parent group (*N* = 400).

Variable	Fathers (*n* = 158) M (SD)	Mothers (*n* = 242) M (SD)	*t*	*p*	*d*
Parental reflexivity (PRF Proxy)	56.60 (16.90)	58.55 (17.46)	−1.10	0.270	−0.11
Aggression index (AI)	47.96 (14.76)	49.34 (14.19)	−0.94	0.347	−0.10
Empathy (EMP)	95.58 (19.02)	92.17 (20.52)	1.67	0.096	0.17
Latent violence index (ILV)	61.66 (21.83)	63.67 (21.07)	−0.92	0.361	−0.09
Uyat intensity (UIS)	34.82 (9.08)	34.91 (9.29)	−0.10	0.922	−0.01
Family-of-origin violence history (FOV, %)	44.9%	42.1%	χ^2^ = 0.20	0.655	–

PRF, parental reflexivity (an operational proxy for parental reflective functioning); ILV, Index of Latent Violence; AI, aggression index; EMP, empathy; UIS, Uyat Intensity Scale; FOV, history of violence in the family of origin. For FOV, the χ^2^ test is reported. *p* < 0.05; *p* < 0.01; *p* < 0.001.

### 4.2 Distribution of reflective functioning among fathers and mothers

The histogram in Figure 1 provides a descriptive view of the PRF distributions. The 40–60-point range comprised 46.8% of fathers compared with 38.8% of mothers, while the proportion with PRF > 70 was 17.7% among fathers and 24.0% among mothers. These distributional features are descriptive and should not be interpreted as statistically established group differences. Levene’s test revealed no statistically significant evidence of unequal variances between fathers and mothers (*F* = 0.10; *p* = 0.750). This result pertains specifically to variance and does not establish equivalence of the overall distributions.

**Figure 1:**
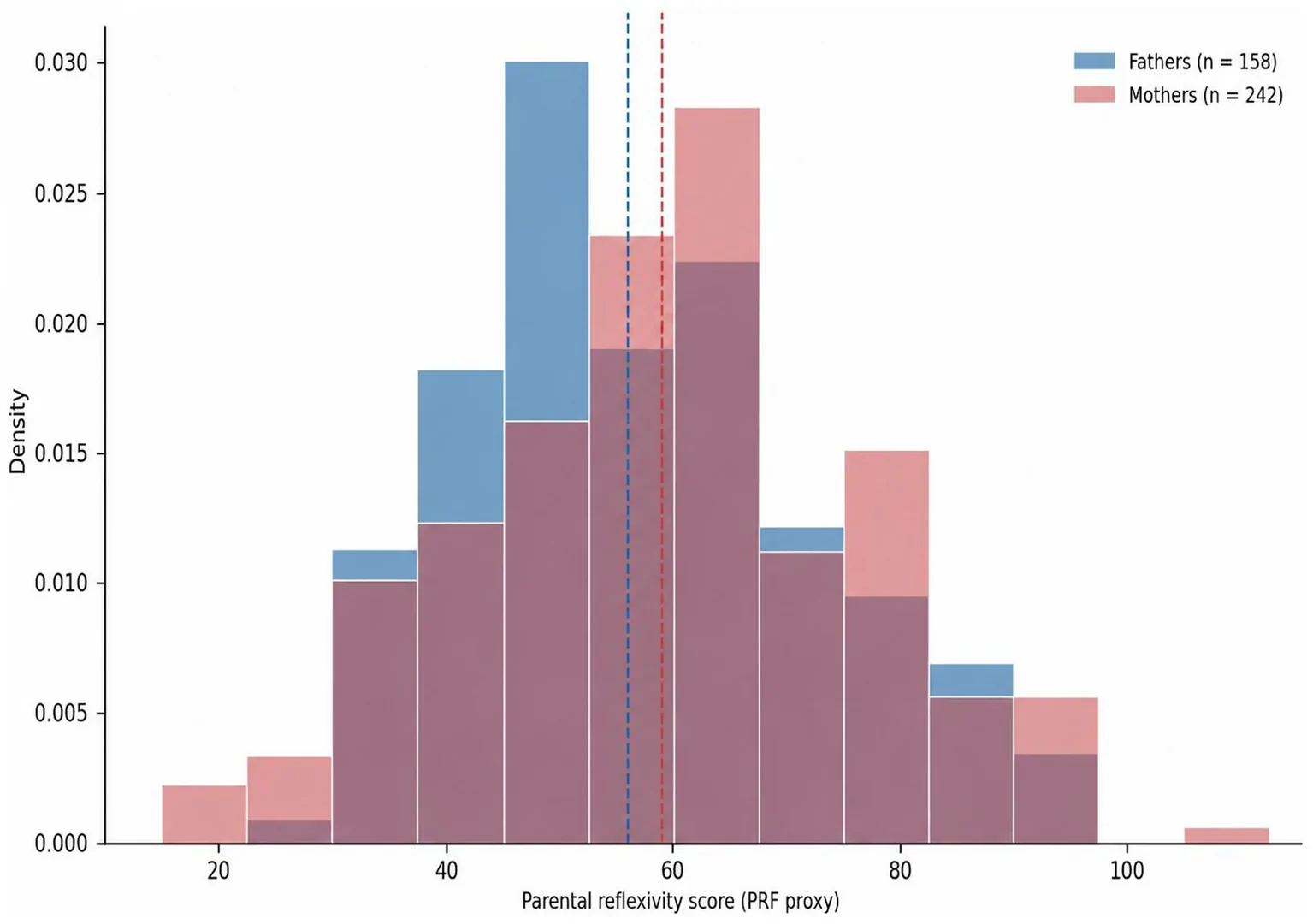
Distribution of parental reflexivity scores among fathers and mothers (*N* = 400).

The median among fathers (56.0) was slightly lower than among mothers (59.0), with similar interquartile ranges (22.0 vs. 22.8). These descriptive summaries suggest some differences in the concentration of scores across the observed distributions, but they do not establish a statistically significant distributional difference. Figure 1 is therefore interpreted descriptively rather than as evidence of a gender-specific distributional pattern.

### 4.3 Association of family-of-origin violence with fathers’ reflexivity

Table 5 compares the psychological profile of fathers according to the presence or absence of a history of violence in their own childhood. The difference in mean levels of reflexivity is 10.37 points in favor of the group without FOV (61.26 versus 50.89; *t* = 4.02; *p* < 0.001; *d* = 0.64), representing a medium-sized effect. Importantly, the parallel analysis among mothers revealed a difference of comparable magnitude and slightly greater size: 63.70 versus 51.48 (*d* = 0.74). Thus, the expectation of a stronger FOV-related reduction in reflexivity specifically among fathers does not receive empirical support: FOV was associated with lower reflexivity among both fathers and mothers. Among fathers, this reduction coincided descriptively with the underrepresentation of highly reflective profiles; however, the mean reflexivity values in the FOV groups were very similar (*M* = 50.89 among fathers and *M* = 51.48 among mothers).

**Table 5:** Psychological profile of fathers by experience of violence in the family of origin (*n* = 158).

Indicator	Without FOV (*n* = 87)	With FOV (*n* = 71)	*t*	*p*	*d*
Parental reflexivity (PRF Proxy)	61.26 (16.14)	50.89 (16.14)	4.02	<0.001	0.64
Latent violence index (ILV)	55.01 (19.98)	69.82 (21.35)	−4.49	<0.001	−0.72
Aggression index (AI)	43.26 (12.88)	53.70 (14.95)	−4.71	<0.001	−0.75
Uyat intensity (UIS)	32.38 (8.59)	37.80 (8.81)	−3.90	<0.001	−0.62
Empathy (EMP)	101.17 (19.19)	88.72 (16.49)	4.32	<0.001	0.69

FOV, history of violence in the family of origin (Family-of-Origin Violence). ILV, Index of Latent Violence; AI, aggression index; UIS, Uyat Intensity Scale; EMP, empathy. *p* < 0.05; *p* < 0.01; *p* < 0.001.

The uyat index was also higher among fathers with FOV than among fathers without FOV (37.80 versus 32.38; *d* = −0.62). This co-occurrence is consistent with, but does not demonstrate, a possible relationship between childhood violence, social shame, and reflective processing. Because the data are cross-sectional, no directional mechanism can be inferred. Figure 2 visualizes the shift in the two PRF distributions by FOV status within the father subsample.

**Figure 2:**
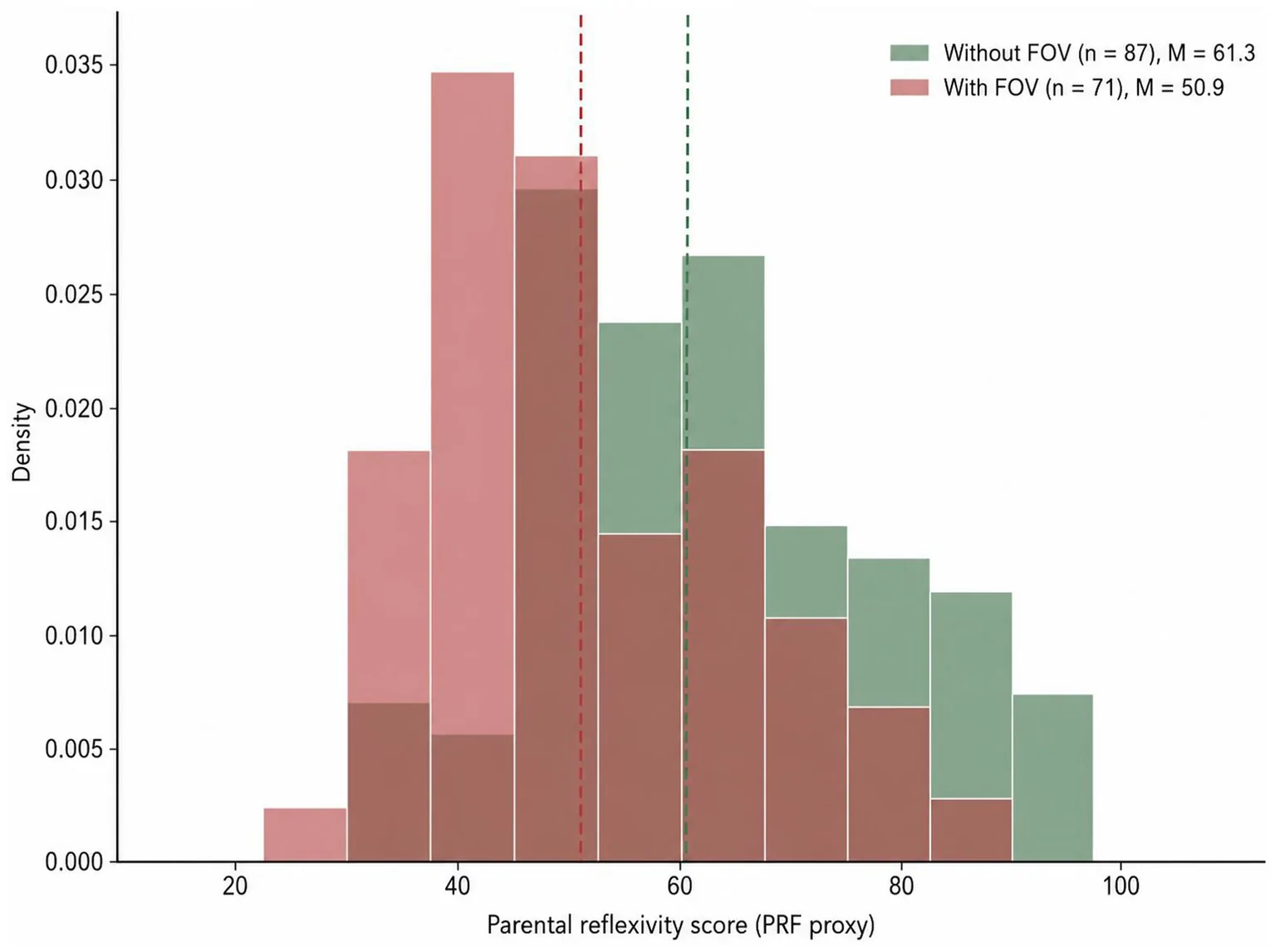
Fathers’ reflexivity by family-of-origin violence (FOV) status (*n* = 158).

### 4.4 Comparative regression analysis in father and mother subsamples

The central analytical result is the comparison of the simple regression coefficients for PRF → ILV in the two subsamples. Among fathers, *b* = −0.900 (SE = 0.074; *t* = −12.13; *p* < 0.001; *R*^2^ = 0.485); among mothers, *b* = −0.779 (SE = 0.060; *t* = −13.09; *p* < 0.001; *R*^2^ = 0.417). The difference between the coefficients amounts to 15.5% in relative terms, but does not reach statistical significance: *z* = −1.27; *p* = 0.204 according to the slope difference test (Paternoster et al., 1998). Stratified bootstrapping yielded a 95% confidence interval for the difference of [−0.320; 0.067], which includes zero; including recruitment-site fixed effects did not change the pattern (*z* = −1.24; *p* = 0.215). Accordingly, H1 is not supported with respect to statistical confirmation of the difference: the steeper slope among fathers should be qualified as a descriptive exploratory pattern, the direction of which is consistent with the theoretical expectation outlined above, but requires verification in substantially larger samples.

Figure 3 presents the father- and mother-specific fitted regression lines with 95% confidence bands over the central PRF score range displayed in the figure. The bands overlap substantially across much of this range, consistent with the nonsignificant direct test of the slope difference. The figure is therefore intended to visualize the estimated associations and their uncertainty rather than to emphasize point predictions in sparsely populated tails of the PRF distribution.

**Figure 3:**
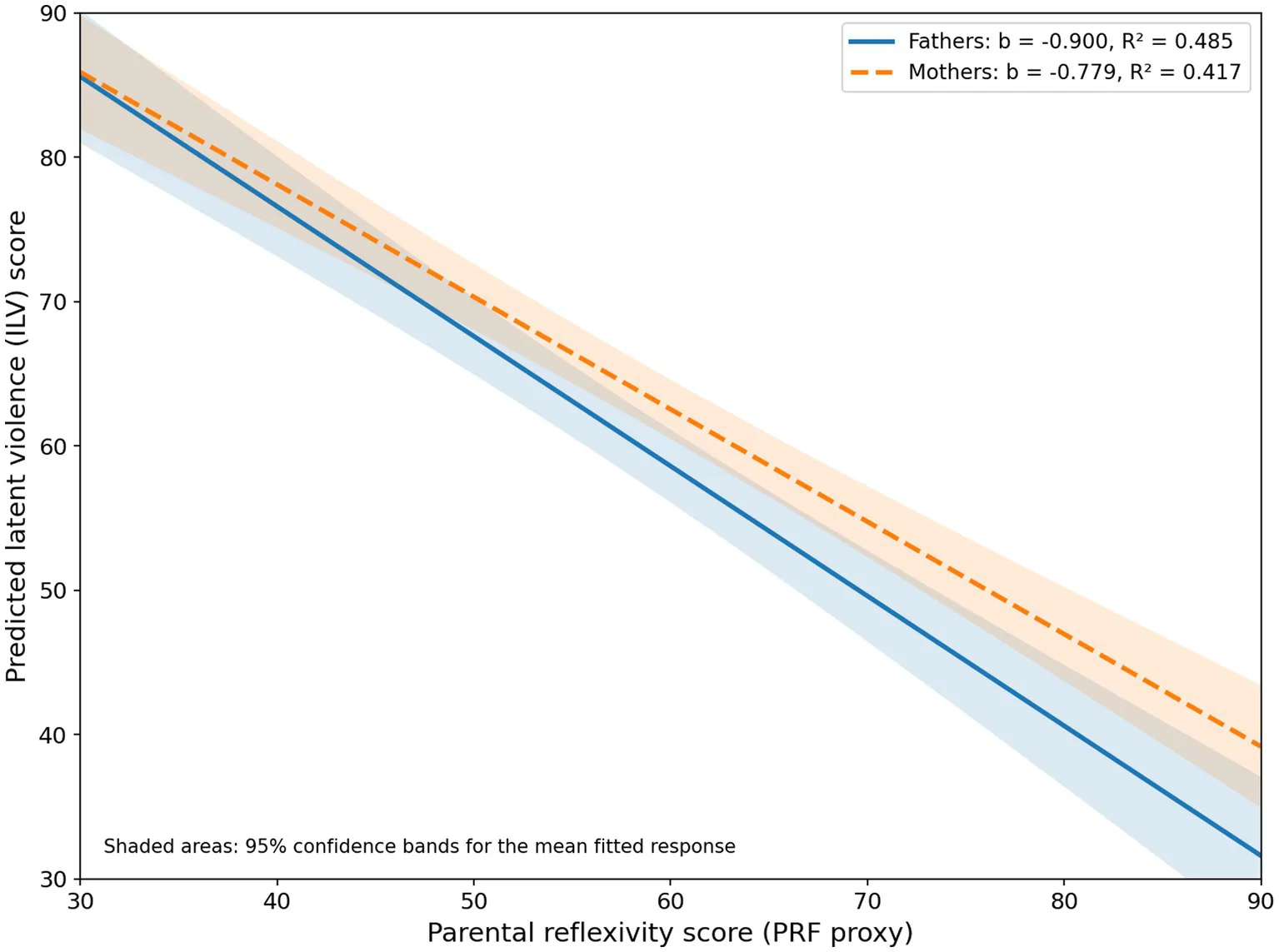
PRF → ILV regressions with 95% confidence bands in father and mother subsamples (slope difference n.s.: *z* = −1.27, *p* = 0.204).

### 4.5 Hierarchical moderated regression: the PRF × parent-group interaction

Table 6 presents the results of a four-step moderation regression model with ILV as the dependent variable. At Step 1, age and education jointly explained less than 0.1% of the variance in ILV in this sample (*R*^2^ = 0.001; *p* = 0.966). This nonsignificant block should not be interpreted as evidence that sociodemographic factors are irrelevant or equivalent across groups. The inclusion of the main predictors at Step 2 produced a substantial increase in explained variance (Δ*R*^2^ = 0.490; *p* < 0.001): reflexivity had the largest standardized coefficient in the model (β = −0.47; *p* < 0.001). Uyat intensity retained an independent contribution (β = 0.13; *p* = 0.001); FOV (β = 0.11; *p* = 0.004) and empathy (β = −0.15; *p* = 0.003) also entered the model as statistically significant predictors.

**Table 6:** Hierarchical moderated regression predicting ILV (*N* = 400).

Predictor/Step	*b*	SE	β	*t*	*p* [95% CI]
Step 1: Demographic control variables					*R*^2^ = 0.001; *F* = 0.03; *p* = 0.966
Age	−0.04	0.16	−0.01	−0.23	0.818 [−0.35; 0.28]
Education	−0.13	1.09	−0.01	−0.11	0.909 [−2.28; 2.02]
Step 2: Main predictors					*R*^2^ = 0.491; Δ*R*^2^ = 0.490***; *F* = 53.95
PRF (centered)	−0.58	0.06	−0.47	−8.98	<0.001*** [−0.70; −0.45]
Parent group (Father = 1)	−2.75	1.60	−0.06	−1.71	0.088 [−5.90; 0.41]
Uyat Intensity (UIS)	0.31	0.10	0.13	3.21	0.001** [0.12; 0.50]
FOV (0/1)	4.91	1.68	0.11	2.92	0.004** [1.60; 8.22]
Empathy (EMP)	−0.16	0.05	−0.15	−2.95	0.003** [−0.26; −0.05]
Step 3: + Interaction					*R*^2^ = 0.493; Δ*R*^2^ = 0.002; *F* = 47.49
PRF × Parent group (centered)	−0.12	0.09	−0.05	−1.28	0.201 [−0.30; 0.06]
Step 4: + Recruitment-site fixed effects					*R*^2^ = 0.494; Δ*R*^2^ = 0.001; *F* = 34.48; recruitment-site block: *F*(3, 388) = 0.38, *p* = 0.766
Recruitment site: Bishkek (ref. Almaty)	1.38	2.00	–	0.69	0.491 [−2.55; 5.31]
Recruitment site: Shymkent	1.93	2.02	–	0.95	0.340 [−2.05; 5.91]
Recruitment site: Turkestan	1.76	2.48	–	0.71	0.479 [−3.11; 6.62]
PRF × Parent group (Step 4)	−0.12	0.09	−0.05	−1.27	0.204 [−0.30; 0.06]

PRF was mean-centered before construction of the interaction term. Parent group was coded father = 1 and mother = 0. b, unstandardized coefficient; SE, standard error; β, standardized coefficient; CI, 95% confidence interval for b. At Step 4, recruitment-site fixed effects were added, with Almaty as the reference category; HC3 robust standard errors yielded substantively identical results. *p* < 0.05; *p* < 0.01; *p* < 0.001. **means *p* < 0.01 and ***means *p* < 0.001.

Adding the PRF × parent-group interaction at Step 3 produced an R^2^ increase of 0.002 (*p* = 0.201)—a statistically nonsignificant increment, with a consistently negative coefficient (*b* = −0.12; 95% CI [−0.30; 0.06]). The result was reproduced when recruitment-site fixed effects were included (Step 4: *p* = 0.204; the recruitment-site block as a whole was nonsignificant: *F*(3, 388) = 0.38; *p* = 0.766), when HC3 robust standard errors were used (*p* = 0.227), in the full sample of *n* = 500 (*p* = 0.245), and in the model with parent-group weighting (*p* = 0.259). Thus, there is no statistical basis for concluding that parent group moderates the association between parental reflexivity and ILV. The descriptively steeper slope among fathers should therefore be treated as hypothesis-generating, and its reproducibility requires testing in studies with greater statistical power.

### 4.6 High ILV scores among parents with family-of-origin violence: an exploratory descriptive analysis

Table 7 and Figures 4, 5 summarize the exploratory subgroup analysis. Among highly reflective fathers (PRF > 70; *n* = 28), five reported a history of FOV and none of these five met the operational high-ILV threshold of ILV ≥ 70 (0/5; 95% Wilson CI [0, 43.4%]). Among highly reflective mothers, 1 of 7 parents with FOV met this threshold (14.3%; 95% Wilson CI [2.6, 51.3%]). The difference was statistically nonsignificant (Fisher’s exact test, *p* = 1.000), and the wide overlapping confidence intervals show that the proportions cannot be distinguished with the available sample. Because the study is cross-sectional and the ILV is an author-developed self-report measure, this analysis does not directly measure intergenerational transmission. It describes the co-occurrence of a reported FOV history and a current ILV score meeting an operational high-score threshold. To assess whether the small-cell pattern depended on the specific cut point, the comparison was repeated using ILV thresholds of ≥ 60, ≥ 65, ≥ 75, and ≥ 80. The father cell remained 0/5 at each threshold; the mother cell was 1/7 at thresholds of 60, 65, and 70 and 0/7 at thresholds of 75 and 80 (Fisher’s exact test, *p* = 1.000 for each comparison). The ILV scores in these two cells were 20, 22, 29, 57, and 58 among fathers and 27, 31, 36, 38, 44, 52, and 72 among mothers. Thus, the descriptive contrast at the operational threshold of 70 is driven by a single mother whose ILV score was 72.

**Table 7:** Mean ILV scores and frequency of high ILV scores (ILV ≥ 70) among parents with FOV by PRF band and parent group (*N* = 400).

Parent group	PRF band	*n*	M ILV (SD)	*n* (FOV = yes)	n high ILV	% high ILV
Fathers	Lower (PRF < 40)	24	82.1 (17.6)	17	14	82.4%
	Middle (PRF 40–70)	106	63.1 (18.2)	49	22	44.9%
	Higher (PRF > 70)	28	38.7 (17.3)	5	0	0.0%
Mothers	Lower (PRF < 40)	36	83.3 (17.7)	27	21	77.8%
	Middle (PRF 40–70)	148	66.6 (16.8)	68	35	51.5%
	Higher (PRF > 70)	58	44.0 (17.1)	7	1	14.3%

The high-ILV frequency is the proportion of parents with FOV = yes whose ILV score was ≥ 70. In this manuscript, ILV ≥ 70 is treated as an operational threshold for exploratory subgroup description, not as a clinically validated cutoff. The high-PRF father–mother comparison was statistically nonsignificant (Fisher’s exact test, *p* = 1.000); 95% Wilson CI: 0/5 –[0, 43.4%]; 1/7 –[2.6, 51.3%]. PRF = parental reflexivity proxy; lower band < 40; middle band 40–70 inclusive; higher band > 70.

**Figure 4:**
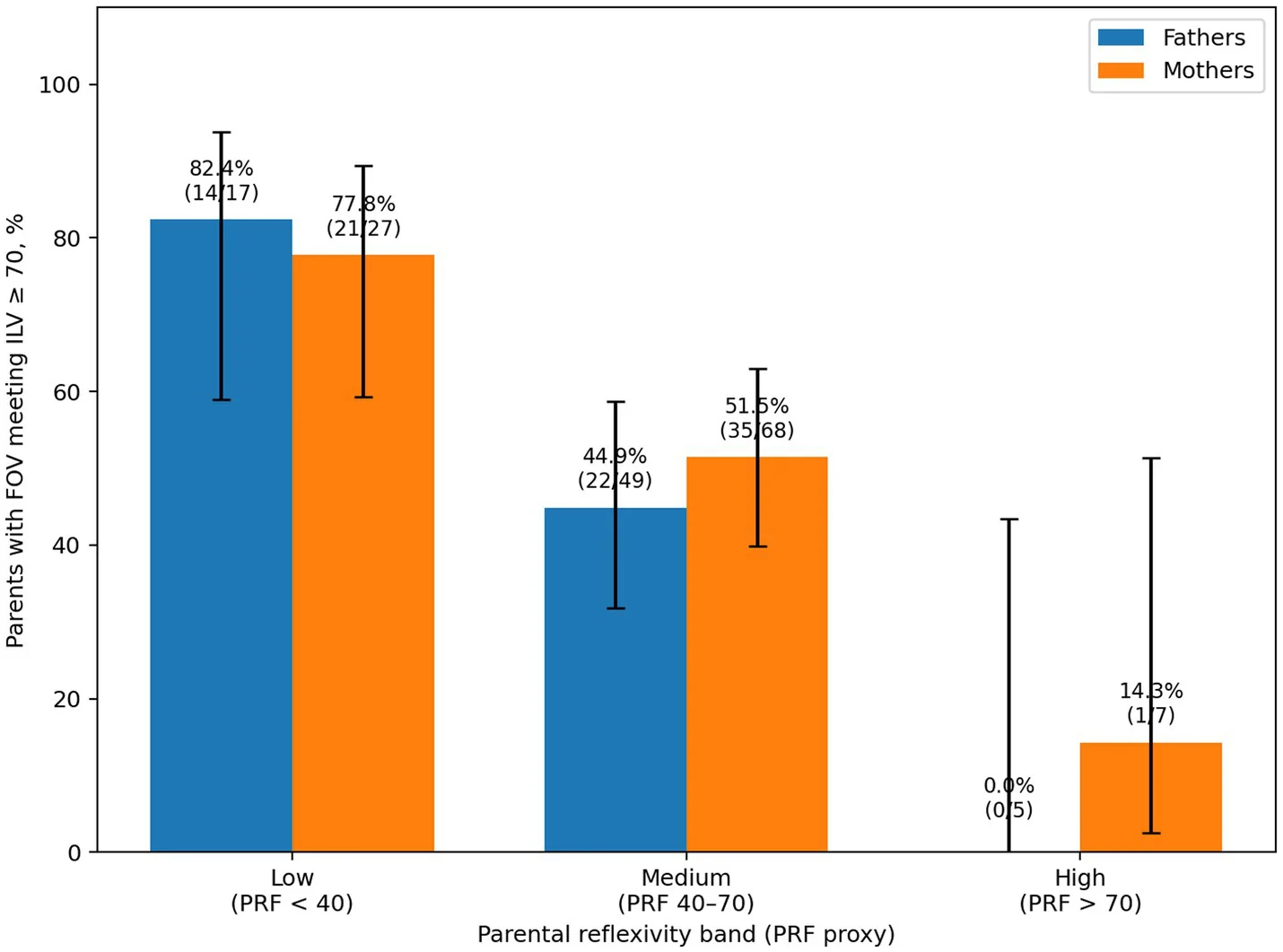
Frequency of ILV ≥ 70 among parents with FOV by reflexivity band and parent group, with 95% Wilson CIs (*N* = 400).

**Figure 5:**
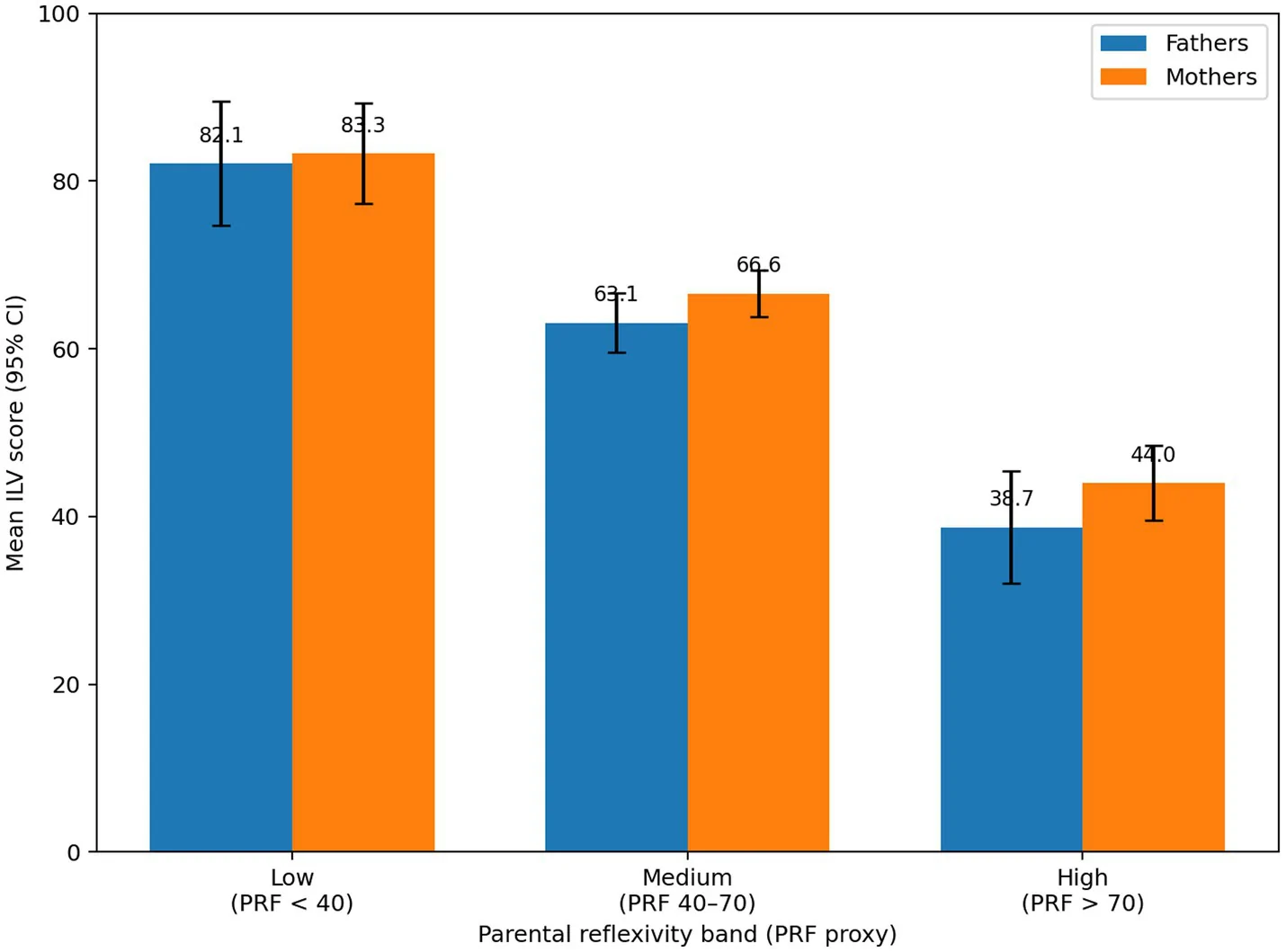
Mean latent-violence (ILV) scores by reflexivity band and parent group, with 95% CIs (*N* = 400).

Figure 4 presents the frequencies of ILV ≥ 70 among parents with FOV across the six descriptive PRF-by-parent-group cells, with 95% Wilson confidence intervals. The point estimates decline across increasing PRF bands in both parent groups. Among fathers, the estimate is 82.4% in the lower band, 44.9% in the middle band, and 0.0% in the higher band; among mothers, the corresponding estimates are 77.8, 51.5, and 14.3%. The confidence intervals are wide in the smallest cells, especially in the higher PRF band, and the between-group pattern should therefore be interpreted descriptively rather than as evidence of differential transmission or protection.

Figure 5 presents mean ILV scores across the same PRF bands with 95% confidence intervals. Mean ILV was lower in the higher PRF band than in the lower PRF band for both fathers and mothers. At the higher PRF band, the means were 38.7 for fathers and 44.0 for mothers, whereas at the lower PRF band they were 82.1 and 83.3, respectively. These descriptive contrasts are consistent with the overall inverse PRF–ILV association, but they do not establish a parent-group difference or a causal effect of reflexivity.

### 4.7 The association between uyat and parental reflexivity

The correlation between PRF and UIS among fathers was *r*s = −0.489 (*p* < 0.001), indicating a significant association of moderate strength. Among mothers, the corresponding correlation was *r*s = −0.440 (*p* < 0.001), and the difference between the correlations was statistically nonsignificant (*z* = −0.60; *p* = 0.546). Accordingly, the present data provide no statistical evidence that the uyat–reflexivity association differs between fathers and mothers. Regression analysis showed that UIS retained an independent contribution to the prediction of ILV (β = 0.13; *p* = 0.001) after controlling for PRF. This finding is consistent with recent Kazakhstani research indicating that parental attitudes and family emotional dynamics are shaped by sociodemographic and family-context factors (Bapayeva et al., 2026). Among fathers with UIS > 45, the mean PRF score was 41.6 (SD = 12.3; *n* = 19), whereas among fathers with UIS < 25 it was 71.3 (SD = 18.7; *n* = 18). These descriptive results are compatible with a possible cultural association between uyat and reflective openness, but the cross-sectional design does not establish a causal mechanism.

Among unmarried fathers (*n* = 9), the mean PRF score was 41.0, while the lowest city-level mean within the father subsample was observed in Bishkek (*M* = 54.3, SD = 14.3), followed by Almaty (*M* = 57.6, SD = 17.0). Because these subgroup comparisons involve small or uneven group sizes and were not designed to identify causal contextual effects, they should be interpreted cautiously. In particular, the present design does not permit country effects to be separated cleanly from city effects because Kyrgyzstan is represented only by Bishkek.

### 4.8 Sensitivity analyses: full sample (*n* = 500), recruitment-site fixed effects, parent-group weighting, and additional family-context covariates

To assess the robustness of the results to the decision to form the analytical subsample of *N* = 400, all key models were re-estimated in the full dataset of n = 500 (158 fathers, 342 mothers). The estimates were reproduced with a high degree of precision (Table 8). In the full sample, the PRF → ILV coefficient among mothers was *b* = −0.782 (SE = 0.048; *R*^2^ = 0.440), compared with *b* = −0.779 in the analytical subsample; the coefficient among fathers was identical by design (*b* = −0.900). The difference between the slopes remained statistically nonsignificant: *z* = −1.34; *p* = 0.181; 95% bootstrap CI for the difference [−0.298; 0.055]. The PRF × parent-group interaction in the hierarchical model with recruitment-site fixed effects was also nonsignificant (*b* = −0.10; *p* = 0.245), including when estimated using weighted least squares with post-stratification weights by parent group (*b* = −0.10; *p* = 0.259; HC3 robust standard errors).

**Table 8:** Sensitivity analysis: key estimates in the analytical subsample (*N* = 400) and the full sample (*n* = 500).

Indicator	*N* = 400	*n* = 500	*n* = 500 (WLS, weights by parent group)
b (PRF → ILV), fathers (SE)	−0.900 (0.074)	−0.900 (0.074)	–
b (PRF → ILV), mothers (SE)	−0.779 (0.060)	−0.782 (0.048)	–
Slope difference: z(p)	−1.27 (0.204)	−1.34 (0.181)	–
95% bootstrap difference	[−0.320; 0.067]	[−0.298; 0.055]	–
PRF × Parent-group interaction: b (p)	−0.12 (0.204)	−0.10 (0.245)	−0.10 (0.259)
Block of recruitment-site fixed effects: F(p)	0.38 (0.766)	0.61 (0.611)	–
High-ILV frequency (ILV ≥ 70) in the higher-PRF subgroup: fathers vs. mothers	0/5 vs. 1/7; *p* = 1.000	0/5 vs. 2/16; p = 1.000	–

The interaction was estimated in the Step 4 specification, with recruitment-site fixed effects included. For WLS, *p*-values are reported using HC3 robust standard errors. High-ILV frequencies (ILV ≥ 70 among parents with FOV) were compared using Fisher’s exact test. Bootstrapping was stratified, with 5,000 replications.

The exploratory high-ILV pattern was also reproduced in the full sample: among highly reflective fathers with FOV, none of five met ILV ≥ 70, whereas among highly reflective mothers with FOV, 2 of 16 met the threshold (12.5%; 95% Wilson CI [3.5, 36.0%]); the difference was statistically nonsignificant (Fisher’s exact test, *p* = 1.000). These threshold-based frequencies are descriptive and should not be interpreted as direct estimates of intergenerational transmission. Recruitment-site fixed effects were nonsignificant in the full-sample specification [*F*(3, 488) = 0.61; *p* = 0.611]. However, nonsignificant city coefficients do not establish the absence of intercity or country-level heterogeneity, and country and city effects cannot be separated cleanly because Kyrgyzstan is represented only by Bishkek. The full-sample and parent-group-weighted sensitivity analyses indicate that excluding 100 mother observations when forming the analytical subsample did not materially alter the main coefficient estimates; they do not resolve questions of country-level generalizability.

An additional sensitivity model examined whether the PRF–ILV association, or the descriptive father–mother difference in its slope, could be explained by current family circumstances not captured by age and education. Model 4 added registered marital status (registered marriage vs. all other statuses), self-rated family income (two dummy variables with “average” as the reference category), and number of children to Model 3 while retaining the PRF × parent-group interaction and recruitment-site fixed effects (Table 3). The four added covariates did not improve the model as a block: *F*(4, 384) = 1.66, *p* = 0.159, although R^2^ increased from 0.494 to 0.503. None of the added covariates reached conventional statistical significance: registered marriage, *b* = 0.94, *p* = 0.557; above-average income, *b* = 2.87, *p* = 0.152; below-average income, *b* = −0.71, *p* = 0.706; and number of children, *b* = −1.71 per additional child, *p* = 0.059.

The main estimates remained substantively unchanged. Parental reflexivity retained the largest coefficient in absolute terms (*b* = −0.53, SE = 0.07, β = −0.42, *p* < 0.001), while the coefficients for uyat intensity (*b* = 0.31, *p* = 0.001), FOV (*b* = 5.01, *p* = 0.003), and empathy (*b* = −0.16, *p* = 0.003) were very similar to those in Model 3. The PRF × parent-group interaction remained nonsignificant, *b* = −0.15 (SE = 0.09), 95% CI [−0.33, 0.04], *p* = 0.117; using HC3 robust standard errors yielded *p* = 0.138. Coding all five marital-status categories separately produced the same substantive pattern (interaction *b* = −0.16, *p* = 0.098). The coefficient for widowed status, based on only 15 participants, reached *p* = 0.047 (*b* = 8.16) and is not interpreted substantively because of the small cell and exploratory multiple testing. In the full sample of *n* = 500, the interaction with the expanded covariate set was also nonsignificant (*b* = −0.12, *p* = 0.152). Thus, the absence of statistically detectable parent-group moderation was robust to the expanded covariate set. Parent-group-specific regressions adjusted for age, education, marital status, income, and number of children yielded slopes of *b* = −0.91 (SE = 0.08) among fathers and *b* = −0.78 (SE = 0.06) among mothers, closely matching the unadjusted estimates reported in section 4.4.

### 4.9 Final status of the hypotheses

H1 did not receive statistical support: the PRF → ILV slope among fathers was descriptively steeper than among mothers, but the direct slope difference was nonsignificant (*z* = −1.27; *p* = 0.204), as was the PRF × parent-group interaction across all specifications, including the model with additional family-context covariates (*p* = 0.117–0.259). H2 was supported: FOV was associated with lower reflexivity in both fathers and mothers; the observed effect sizes were *d* = 0.64 and *d* = 0.74, respectively, providing no evidence of a stronger association among fathers. H3 remains a preliminary descriptive observation: none of five highly reflective fathers with FOV met the operational ILV ≥ 70 threshold, compared with 1 of 7 mothers, but the difference was statistically nonsignificant (*p* = 1.000) and does not constitute evidence of intergenerational transmission or its absence. H4 was not supported with respect to a parent-group difference: the uyat–reflexivity association was significant among fathers (*r*ₛ = −0.489; *p* < 0.001) and mothers (*r*ₛ = −0.440; *p* < 0.001), but the two correlations did not differ statistically (*z* = −0.60; *p* = 0.546).

## 5 Discussion

### 5.1 Parental reflexivity and latent psychological violence

The most robust finding of the present study is the strong inverse association between parental reflexivity and latent psychological violence among both fathers and mothers. Although the estimated slope was descriptively steeper among fathers (*b* = −0.900) than among mothers (*b* = −0.779), neither the direct comparison of coefficients nor the PRF × parent-group interaction provided statistically significant evidence that this association differs between the two parent groups. Accordingly, the present data do not establish a father-specific protective effect of reflexivity. This conclusion remained unchanged after marital status, self-rated family income, and number of children were added to the model, indicating that the nonsignificant parent-group interaction was not sensitive to the expanded covariate set.

The descriptively different slopes may nevertheless be useful for hypothesis generation, particularly because fathers remain comparatively underrepresented in research on parental reflexivity. The same caution applies to the subgroup of highly reflective parents with FOV: the observed frequency was 0/5 among fathers and 1/7 among mothers, but the difference was statistically nonsignificant and the confidence intervals were wide. These observations therefore identify questions for future adequately powered research rather than evidence of a gender-specific mechanism.

Potential explanations based on gendered emotional socialization, social support, help-seeking, or cultural expectations remain theoretical propositions rather than empirical findings of the present study because these mechanisms were not directly measured. Future research should assess such factors explicitly before attributing descriptive differences between fathers and mothers to gender-specific compensatory resources.

### 5.2 Family-of-origin violence and parental reflexivity

A history of violence in the family of origin was associated with substantially lower reflexivity among both fathers and mothers. The estimated difference was 10.37 points among fathers (*d* = 0.64) and 12.22 points among mothers (*d* = 0.74). Thus, the present data do not support a stronger FOV-related reflexivity deficit among fathers. Rather, they indicate that FOV is associated with lower parental reflexivity across both parent groups. Potential gender-specific differences in the social or cultural context surrounding this association remain plausible theoretical questions, but they were not directly measured in the present study.

Stress-response dysregulation and difficulties in emotion regulation have been proposed as possible pathways linking childhood adversity with later behavioral regulation (Yehuda and Lehrner, 2018; Teicher et al., 2012). However, neurobiological processes were not measured in the present study, and no mechanism can be inferred from the cross-sectional associations reported here. Longitudinal and multimethod studies are needed to test whether such processes contribute to the association between FOV and parental reflexivity and whether any pathways differ between fathers and mothers.

### 5.3 High ILV scores among highly reflective parents with FOV: exploratory interpretation

The observation that none of the five highly reflective fathers with FOV met the operational ILV ≥ 70 threshold is potentially informative but highly uncertain. The 95% Wilson confidence interval for the zero proportion extends to 43.4%, and Fisher’s exact test shows no statistically detectable difference from mothers (*p* = 1.000). Moreover, ILV ≥ 70 has not been established here as a clinical diagnostic cutoff, and the cross-sectional design does not directly observe transmission of behavior from one generation to the next. The result should therefore be described only as a small-cell, threshold-based pattern that may motivate future longitudinal research.

Mentalization and reflective-functioning theories provide a plausible conceptual basis for expecting reflexivity to be associated with less reactive parenting behavior (Slade, 2005; Siegel, 2012). However, the present study did not measure the proposed moment-to-moment “reflective interval,” behavioral responses to children, or narrative restructuring. These mechanisms therefore remain theoretical explanations to be tested rather than empirical findings of the current study.

### 5.4 Practical implications

The present cross-sectional findings do not establish that increasing parental reflexivity will causally reduce latent psychological violence, nor do they identify differential treatment effects for fathers and mothers. Nevertheless, the robust inverse association between reflexivity and ILV suggests that reflexivity is a relevant construct for further evaluation in prevention research. Future intervention studies could examine whether reflective-parenting components are effective for parents with a history of family-of-origin violence and whether father-inclusive adaptations improve engagement or outcomes. In the Central Asian context, culturally adapted approaches that address uyat-related concerns and norms surrounding emotional expression may also be worthy of prospective evaluation.

At the level of family and educational services, the present findings support further evaluation of parental reflexivity as a potential screening and intervention target rather than immediate implementation of a father-specific program. Any practical recommendations should be tested prospectively using longitudinal or randomized designs before claims about prevention effectiveness or subgroup-specific benefit are made.

## 6 Limitations

The first limitation is the cross-sectional design, which does not permit causal conclusions. The observed association between PRF and ILV may partly reflect unmeasured third variables and reciprocal influences. Longitudinal studies incorporating behavioral observation are needed to clarify temporal ordering and potential causal pathways.

The second limitation concerns the small cells in the factorial analysis and the interpretation of threshold-based subgroup frequencies. The “higher PRF, FOV = yes” father cell included only five participants; the 95% Wilson confidence interval for the observed 0/5 proportion ([0, 43.4%]) is compatible with a wide range of underlying rates. In addition, ILV ≥ 70 is used only as an operational high-score threshold and has not been established as a clinically validated cutoff. Sensitivity checks using alternative thresholds of 60, 65, 75, and 80 did not produce statistically significant father–mother differences, but these checks do not resolve the small-cell problem or validate any threshold clinically. Accordingly, the subgroup frequencies should not be interpreted as direct estimates of intergenerational transmission or as evidence that transmission is absent. Independent external validation of the ILV and its interpretive thresholds is needed.

The third limitation is the self-report nature of all measures. Social-desirability and shame-related response tendencies may contribute to underreporting of psychologically harmful parenting behaviors or uyat-related experiences, and this possibility could not be quantified in the present design. Behavioral observation of parent–child interaction would strengthen future assessments.

The fourth limitation is the urban composition of the sample. All four study sites are administrative centers; rural and small-town populations are not represented, which limits geographic generalizability.

The fifth limitation concerns contextual comparability across sites. Kyrgyzstan is represented only by Bishkek, whereas Kazakhstan is represented by Almaty, Shymkent, and Turkestan; consequently, country and city effects are not separately identifiable. In addition, the current analysis did not establish measurement invariance across country or survey-language groups. Cross-site contrasts therefore should not be interpreted as evidence of national or language-specific differences. Although the survey languages and translation procedure are described in Section 3.1, the language version completed by each participant was not stored in the analytical dataset, so measurement invariance across language versions could not be tested.

The sixth limitation is operational in nature: reflexivity was measured using A. V. Karpov’s Reflexivity Scale—a general metacognitive instrument used in the present study as a proxy indicator of parental reflective functioning—rather than a specialized instrument such as the PRFQ (Luyten et al., 2017). Although the psychometric validation conducted in this study and the established measurement invariance across father and mother groups—metric and partial scalar levels for the ILV, and full scalar invariance for the UIS—strengthen the validity of father–mother comparisons, the substantive conclusions should properly be attributed to parental reflexivity in a broad sense. Direct replication using a validated Central Asian adaptation of the PRFQ remains a task for future research.

The seventh limitation concerns the granularity of available family-context information. The analytical dataset contains marital status, self-rated family income, and number of children, which were incorporated into the sensitivity model in Table 3, but it does not contain child-level age or sex variables, a target-child identifier, or monetary household income. Because parents with multiple eligible children were instructed to report their usual behavior toward their children as a whole, rather than toward one identified child, and because family income was assessed on a three-category scale, residual confounding by child characteristics and socioeconomic circumstances cannot be excluded. Future studies should identify a target child, record the child’s age and sex, and use more differentiated socioeconomic indicators.

## 7 Conclusion

The present exploratory study of 400 urban parents from four cities in Kazakhstan and Kyrgyzstan identified a robust inverse association between parental reflexivity and self-reported latent psychological violence. This association was evident among both fathers and mothers. Although the estimated PRF–ILV slope was descriptively steeper among fathers (*b* = −0.900) than among mothers (*b* = −0.779), the difference between coefficients was not statistically significant (*z* = −1.27; *p* = 0.204), and the PRF × parent-group interaction was nonsignificant across all model specifications. The present data therefore do not establish parent-group moderation or a father-specific protective effect of reflexivity.

A history of violence in the family of origin was associated with lower reflexivity in both parent groups. In the small higher-reflexivity subgroup with FOV, none of five fathers and 1 of 7 mothers met the operational ILV ≥ 70 threshold; this difference was statistically nonsignificant. Because this threshold is not treated as a clinical cutoff and the study is cross-sectional, the result cannot be interpreted as evidence that intergenerational transmission was absent or interrupted. The pattern should be examined in larger, longitudinal, independently replicated samples.

Because the sample was drawn exclusively from four urban centers and Kyrgyzstan was represented only by Bishkek, the findings should not be generalized to the national populations of Kazakhstan or Kyrgyzstan. Country and city effects cannot be cleanly separated in the present design. The cross-sectional and self-report nature of the data also precludes causal conclusions about whether higher reflexivity reduces psychologically harmful parenting behavior.

Future research should use larger and more geographically diverse samples, longitudinal and behavioral designs, direct measurement of family-context and socioeconomic factors, and specialized measures of parental reflective functioning such as culturally validated adaptations of the PRFQ (Luyten et al., 2017). Intervention studies are also needed to determine whether changes in parental reflexivity lead to changes in latent psychological violence and whether intervention effects differ between fathers and mothers.

## Data Availability

The anonymized dataset, codebook, and statistical analysis scripts (Python 3.11; NumPy, SciPy, StatsModels) are available to qualified researchers from the corresponding author upon reasonable request. Given the sensitive nature of the data on family violence, access is provided in a controlled manner that precludes the possibility of participant de-anonymization.

## References

[ref1] Addis M. E. Mahalik J. R. (2003). Men, masculinity, and the contexts of help seeking. Am. Psychol. 58, 5–14. doi: 10.1037/0003-066X.58.1.5, 12674814

[ref2] Akhmetova S. Durrani N. Karabay A. (2025). Intimate partner violence and the culture of shame in Kazakhstan: voices of survivors and violence against women professionals. Violence Against Women 32, 4429–4452. doi: 10.1177/10778012251384563, 41043007

[ref3] Bapayeva S. Bapayeva M. Kuantkanova F. Bagibayeva M. Aubakir A. Baizdrakhmanova A. (2026). Socio-demographic features of parental attitudes. Vestnik KazNPU imeni Abaya. Series: Psychology 87, 327–351. doi: 10.51889/2959-5967.2026.87.2.028

[ref4] Bateman A. Fonagy P. (2004). Psychotherapy for Borderline Personality Disorder. Oxford: Oxford University Press.

[ref5] Bowlby J. (1969). Attachment and Loss: Vol. 1. Attachment. New York, NY: Basic Books.

[ref6] Brody L. R. (1997). Gender and emotion: beyond stereotypes. J. Soc. Issues 53, 369–393. doi: 10.1111/j.1540-4560.1997.tb02448.x

[ref7] Buss A. H. Perry M. (1992). The aggression questionnaire. J. Pers. Soc. Psychol. 63, 452–459. doi: 10.1037/0022-3514.63.3.452, 1403624

[ref8] Buttitta K. V. Smiley P. A. Kerr M. L. Rasmussen H. F. Querdasi F. R. Borelli J. L. (2019). In a father’s mind: paternal reflective functioning, sensitive parenting, and protection against socioeconomic risk. Attach Hum. Dev. 21, 445–466. doi: 10.1080/14616734.2019.1582596, 30990121

[ref9] Chen F. F. (2007). Sensitivity of goodness of fit indexes to lack of measurement invariance. Struct. Equ. Model. 14, 464–504. doi: 10.1080/10705510701301834

[ref10] Cheung G. W. Rensvold R. B. (2002). Evaluating goodness-of-fit indexes for testing measurement invariance. Struct. Equ. Model. 9, 233–255. doi: 10.1207/s15328007sem0902_5

[ref11] Cicchetti D. Toth S. L. (2005). Child maltreatment. Annu. Rev. Clin. Psychol. 1, 409–438. doi: 10.1146/annurev.clinpsy.1.102803.144029, 17716094

[ref12] Efron B. Tibshirani R. J. (1993). An Introduction to the Bootstrap. New York, NY: Chapman & Hall.

[ref13] Eisenberg N. Cumberland A. Spinrad T. L. (1998). Parental socialization of emotion. Psychol. Inq. 9, 241–273. doi: 10.1207/s15327965pli0904_1, 16865170 PMC1513625

[ref14] Fonagy P. Gergely G. Jurist E. L. Target M. (2002). Affect Regulation, Mentalization, and the Development of the Self. New York, NY: Other Press.

[ref15] Fonagy P. Target M. (1997). Attachment and reflective function: their role in self-organization. Dev. Psychopathol. 9, 679–700. doi: 10.1017/S0954579497001399, 9449001

[ref16] Hu L. Bentler P. M. (1999). Cutoff criteria for fit indexes in covariance structure analysis: conventional criteria versus new alternatives. Struct. Equ. Model. 6, 1–55. doi: 10.1080/10705519909540118

[ref17] Kan M. (2023). Are gender attitudes and gender division of housework and childcare related to fertility intentions in Kazakhstan? Genus 79:21. doi: 10.1186/s41118-023-00200-1

[ref18] Karpov A. V. (2003). Reflexivity as a mental property and a method for its diagnosis [in Russian]. Psikhol. Zh. 24, 45–57.

[ref19] Levant R. F. (1992). Toward the reconstruction of masculinity. J. Fam. Psychol. 5, 379–402. doi: 10.1037/0893-3200.5.3-4.379

[ref20] Luyten P. Mayes L. C. Nijssens L. Fonagy P. (2017). The parental reflective functioning questionnaire: development and preliminary validation. PLoS One 12:e0176218. doi: 10.1371/journal.pone.0176218, 28472162 PMC5417431

[ref21] Paternoster R. Brame R. Mazerolle P. Piquero A. (1998). Using the correct statistical test for the equality of regression coefficients. Criminology 36, 859–866. doi: 10.1111/j.1745-9125.1998.tb01268.x

[ref22] Putnick D. L. Bornstein M. H. (2016). Measurement invariance conventions and reporting: the state of the art and future directions for psychological research. Dev. Rev. 41, 71–90. doi: 10.1016/j.dr.2016.06.004, 27942093 PMC5145197

[ref23] Seitakhmetova N. Zhandossova S. (2019). Rol' instituta sem'i v formirovanii kazakhstanskoi identichnosti [in Russian]. Adam Alemi 3, 101–108.

[ref24] Seitakhmetova N. Zhandossova S. (2026). Between security and belonging: state policy on religious extremism, political values and the politics of identity. Front. Polit. Sci. 8:1818270. doi: 10.3389/fpos.2026.1818270

[ref25] Siegel D. J. (2012). The Developing Mind. 2nd Edn New York, NY: Guilford Press.

[ref26] Slade A. (2005). Parental reflective functioning: an introduction. Attach Hum. Dev. 7, 269–281. doi: 10.1080/14616730500245906, 16210239

[ref27] Stover C. S. Kiselica A. (2014). Reflective functioning in fathers: an initial empirical examination. Infant Ment. Health J. 35, 452–461. doi: 10.1002/imhj.21459, 25798495 PMC4710356

[ref28] Stuhrmann L. Y. Göbel A. Bindt C. Mudra S. (2022). Parental reflective functioning and its association with parenting behaviors in infancy and early childhood: a systematic review. Front. Psychol. 13:765312. doi: 10.3389/fpsyg.2022.765312, 35310277 PMC8927808

[ref29] Teicher M. H. Anderson C. M. Polcari A. (2012). Childhood maltreatment is associated with reduced volume in the hippocampal subfields. Proc. Natl. Acad. Sci. USA 109, 17724–17729. doi: 10.1073/pnas.1115396109, 22331913 PMC3295326

[ref30] Thibault H. Caron J.-F. (Eds.) (2022). Uyat and the Culture of Shame in Central Asia. Singapore: Palgrave Macmillan. doi: 10.1007/978-981-19-4328-7

[ref31] Toktarbekova L. Zhandossova S. Bektenova M. (2025). Kazakh women’s visual religious identity and Islamic agency on Instagram. Pharos J. Theol. 106, 1–14. doi: 10.46222/pharosjot.106.3041

[ref32] Wejnert B. Djumabaeva A. (2005). From patriarchy to egalitarianism: parenting roles in democratizing Poland and Kyrgyzstan. Marriage Fam. Rev. 36, 147–171. doi: 10.1300/J002v36n03_08

[ref33] Yehuda R. Lehrner A. (2018). Intergenerational transmission of trauma effects: putative role of epigenetic mechanisms. World Psychiatry 17, 243–257. doi: 10.1002/wps.20568, 30192087 PMC6127768

[ref34] Yusupov I. M. (1995). Psychology of Empathy: Theoretical and Applied Aspects [in Russian]. Doctoral dissertation. Saint Petersburg, Russia: Saint Petersburg State University.

[ref35] Zhandossova S. Seitakhmetova N. Bektenova M. (2023). K voprosu o vliianii islama na semeino-brachnye otnosheniia v kazakhstanskom obshchestve [in Russian]. Kazakhstan Spektr 108, 42–56. doi: 10.52536/2415-8216.2023-4.03

